# The Bilingual Home Language Boost Through the Lens of the COVID-19 Pandemic

**DOI:** 10.3389/fpsyg.2021.667836

**Published:** 2021-07-20

**Authors:** Li Sheng, Danyang Wang, Caila Walsh, Leah Heisler, Xin Li, Pumpki Lei Su

**Affiliations:** ^1^Language Learning and Bilingualism Laboratory, Department of Communication Sciences and Disorders, University of Delaware, Newark, DE, United States; ^2^Department of Communicative Disorders and Sciences, San Jose State University, San Jose, CA, United States

**Keywords:** COVID-19, quarantine, bilingual, Mandarin, English, comprehension, production

## Abstract

Usage-based accounts of language acquisition suggest that bilingual language proficiency is dynamic and susceptible to changes in language use. The COVID-19 pandemic led to unprecedented modifications in the language learning environment of developing bilinguals. Drawing on this unique opportunity, we analyzed existing data of two matched groups of Mandarin-English bilingual children (ages 4 to 8 years, *n* = 38), one tested before (pre-COVID group) and the other during (COVID group) the pandemic. The dataset comprises responses to a language environment questionnaire, and scores on a sentence comprehension task and a sentence recall task in the bilinguals’ two languages. Questionnaire data revealed a richer Mandarin language environment for children in the COVID group compared to peers in the pre-COVID group. On both comprehension and production tasks, the two groups performed comparably in English but the COVID group showed better performance in Mandarin than the pre-COVID group. Within the pre-COVID group, English was stronger than Mandarin in both comprehension and production. Within the COVID group, the two languages were balanced in comprehension and Mandarin was stronger than English in production. Moreover, language use variables were correlated with production performance in both languages. These patterns illustrate the intimate relationships between language use and bilingual language proficiency through the lens of COVID-19 induced language environment modification.

## Introduction

Incremental or usage-based accounts of language acquisition suggest that bilingual language proficiency is dynamic, fluid, and subject to the influences of language use patterns (Wulff and Ellis, 2018; Oppenheim et al., 2020). In developing bilinguals, changes in the language learning environment bring about rather rapid shifts in individuals’ language proficiency profiles. In the case of international adoption, an extreme example of environmental change, the adoptees may quickly lose their original first language and catch up to the norms of the new “first language” in as short as 16 months (Glennen, 2002; Krakow et al., 2005). In the case of study abroad, significant gains in speaking proficiency of the immersion language are reported across studies (Dewey et al., 2012; Di Silvio et al., 2016), and associations are established between the amount of gains and the amount of conversational experience with native speakers of the immersion language (Dewey et al., 2012). In early sequential bilinguals who grow up speaking a home language, shortly after the onset of systematic exposure to the societal language, children begin to change their preferred or dominant language in certain contexts (Kohnert and Bates, 2002; Pham and Kohnert, 2014; Sheng, 2014; Sheng et al., 2014). The so-called “summer vacation effect” provides additional evidence on usage-driven changes in bilingual children’s oral language proficiency (Hammer et al., 2008; Rojas and Iglesias, 2013). Illustrated by longitudinal studies of young Spanish-English bilingual children, this effect suggests that L2 (English) but not L1 (Spanish) growth rates differ between the academic year and the summer months. For instance, over a 3-year span, Rojas and Iglesias (2013) showed linear growth in English expressive language skills during each academic year, but reduced English growth during the summer, suggesting an effect of the lack of systematic support and exposure to English during the summer. In contrast, Spanish growth was not negatively impacted by summer vacation.

The COVID-19 pandemic has led to stay-at home orders worldwide. This resulted in abrupt changes in many bilingual children’s language environment. With schools and daycares closed and peer social interactions attenuated, the amount of English use may have diminished in countries such as the United States where English is the majority language. At the same time, use of the home language may have increased as family members became the primary or only conversational partners. There is both anecdotal (Hardach, 2020) and emerging research evidence (Serratrice, 2020) that quarantine was associated with “a return to native languages” in bilingual children—reduced use of the once-dominant societal language, accompanied by more use of and shifted preference for the home language. Increased opportunities to speak and hear the home language was especially noticeable in preschool and primary school age children (Serratrice, 2020). The current study aims to provide more evidence on how this unprecedented environmental modification affects language use and proficiency of bilingual children. Specifically, we took advantage of this natural experiment by examining available data of matched samples of bilingual children who were tested before and during the pandemic.

Before posing our questions, it is important to point out that the testing modes are different between the two samples by necessity: one group was tested in person, the other remotely *via* video chat. However, emerging evidence suggests that the two testing modes are largely comparable in terms of child engagement, speech intelligibility, amount and characteristics of language elicited, and reliability of transcription and scoring (Waite et al., 2010; Ciccia et al., 2011; Sutherland et al., 2017; Manning et al., 2020). It is also important to point out that the current comparisons are cross-sectional rather than longitudinal. To tease apart the effect of modified environment (i.e., quarantine) from the effect of development, two matched samples are required—one undergoes quarantine and the other does not; and both samples should be assessed at two time points—once before and once during the pandemic. This design was impossible to achieve given the unpredictability of the pandemic. Nevertheless, our dataset contains comparable samples of bilingual children drawn from a relatively homogeneous bilingual population, information on children’s language use patterns, and language performance data in comprehension and production modalities in both languages. Thus, this dataset allows us to answer the following questions:

Do language use patterns differ between bilingual children tested before and during the pandemic?
Does language performance differ between bilingual children tested before and during the pandemic?
Are language use and bilingual language performance related to each other?

Based on available evidence, we predicted that English language use would be more limited but home language use would be greater and richer in the children undergoing quarantine than in their peers tested before the pandemic. In addition, English language performance would be weaker but home language performance would be stronger in children undergoing quarantine. Finally, language use and language performance would be related.

## Materials and Methods

### Participants

The participants were selected from an ongoing study that aims to develop and validate a set of oral language measures for Mandarin-English bilingual children between three and 10 years of age (*n* = 102). Eleven children were removed from the dataset because they were in a Mandarin immersion program but did not use Mandarin at home (*n* = 7), had a known disorder such as hearing impairment and ADHD (*n* = 2), or were enrolled in the study twice (*n* = 2). Of the 91 remaining children, 40 were tested between January and August of 2019 and 51 were tested between June 2020 and April 2021. We performed a two-step process to select the participants for the current study. First, we screened the data and removed participants if there were missing data on any of the five main instruments: the language environment questionnaire, the English comprehension task, the Mandarin comprehension task, the English production task, and the Mandarin production task. A total of 22 children tested in 2019 and 44 children tested in 2020–21 remained eligible after data screening. Next, each eligible child from the 2019 cohort was matched to a child from the 2020–21 cohort using three criteria: (1) within 6 months on chronological age, (2) within one point on maternal education level (see Table 1 for information), and (3) within 12 months for duration of systematic English exposure. Biological sex was not a matching criterion. This resulted in 19 pairs of matched participants. To re-iterate, 19 children were assessed between January 28th, 2019 and August 3rd, 2019 and were hence included in the pre-COVID group. These children resided in the states of California, Delaware, and Pennsylvania at the time of testing. The remaining 19 children were assessed between June 5th, 2020 and January 25th, 2021 and were included in the COVID group. These children resided in the states of California, Delaware, Florida, Massachusetts, Pennsylvania and Texas, as well as Canada. At the time of testing, nine of the 19 COVID group children were on summer break, six were schooled online, two were schooled using a hybrid mode, one went to in-person school, and one child was not in school. Schooling mode information was not collected for children in the pre-COVID group. In-person schooling was predominant in the United States prior to COVID.

**TABLE 1:** Participant information and language use.

	Pre-COVID Mean (SD)	COVID Mean (SD)	Group difference *t*-test or Mann-Whitney U test
Age in months	77.00 (14.30)	77.37 (15.86)	*t*(35.62) = −0.08, *p* = 0.94, *d* = 0.02
Sex	8F;11M	13F;6M	
Duration of English exposure (in months)	49.58 (16.98)	49.00 (15.68)	*t*(35.77) = 0.11, *p* = 0.91, *d* = 0.04
Maternal education	4.84 (0.50)	4.84 (0.37)	*W* = 188.50, *p* = 0.71
Non-verbal IQ	118.06 (16.18)	116.26 (25.70)	*W* = 142.50, *p* = 0.77
English proficiency rating	4.42 (0.53)	4.26 (0.62)	*W* = 205.00, *p* = 0.48
Mandarin proficiency rating	4.22 (0.56)	4.31 (0.48)	*W* = 175.00, *p* = 0.88
Language use in the home	0.32 (0.22)	0.20 (0.16)	*W* = 123.50, *p* = 0.10
English richness	0.71 (0.16)	0.69 (0.19)	*W* = 175.00, *p* = 0.87
Mandarin richness	0.25 (0.16)	0.49 (0.22)	*W* = 74.00, *p* = 0.001

*Maternal education was reported on a 5-point scale: 1 = elementary school, 2 = secondary school, 3 = some college, 4 = Bachelor’s degree, 5 = Master’s or higher. Non-verbal IQ was reported in standard scores using the Primary Test of Non-verbal Intelligence (Ehrler and McGhee, 2008). Non-verbal IQ scores were missing for three children in the Pre-COVID group. English and Mandarin proficiency was rated by the primary caregiver using a 0–5 scale that collected ratings of the child’s oral language in the domains of vocabulary, sentence length, speech intelligibility, listening comprehension, and grammatical proficiency (adapted from the Inventory to Assess Language Knowledge; Peña et al., 2018). Scores across the five domains were averaged to obtain an overall proficiency rating for each language. Language use at home varies from 0 to 1, with 0.5 meaning equal English and Mandarin use at home, values > 0.5 meaning more English than Mandarin, and values < 0.5 meaning more Mandarin than English. Language richness scores vary from 0 to 1, with higher scores meaning richer language use.*

As seen in Table 1, the two groups were closely matched on age, maternal education, and duration of English exposure. The two groups were also highly similar on non-verbal intelligence and English and Mandarin proficiency as rated by primary caregivers. Both parents of all 38 children identified Mandarin as their mother tongue (i.e., language spoken when growing up). Caregivers’ self-rated spoken English fluency was also comparable between groups. Specifically, on a five-point scale, with 0 meaning no speaking ability and 4 meaning very fluent in English, the average fluency for mothers were 3.05 (*SD* = 0.91) for the pre-COVID group and 3.05 (*SD* = 0.87) for the COVID group, *p* = 0.93. The average for fathers was 3.26 (*SD* = 0.93) for the pre-COVID and 3.32 (*SD* = 0.60) for the COVID group, *p* = 0.80. Individual level data are presented in the Supplementary Materials. The study was approved by the Institutional Review Board of the University of Delaware. Parents signed an informed consent and children gave verbal assent before testing began.

### Materials

Testing materials included instruments to measure children’s language comprehension and production, language environment, ratings of children’s proficiency, and non-verbal intelligence.

Language comprehension and production were measured with a sentence comprehension task and a sentence recall task. The English sentence comprehension task includes 36 items assessing noun plurals, prepositions, quantifiers, passive sentences, and relative clauses. The Mandarin sentence comprehension task includes 44 items assessing classifiers, prepositions, quantifiers, passive sentences, and relative clauses. The comprehension stimuli were taken from the Mandarin English Receptive Language Screener (MERLS, Sheng and Wang, unpublished; see Supplementary Material “MERLS test items” for design considerations and sample items), a tool currently under development in our laboratory. Reliability and concurrent validity of the MERLS were assessed in a subsample of the larger cohort of children who completed additional testing. Test-retest reliability of the MERLS is.92. Concurrent validity is.60 with the Test for Reception of Grammar-2nd Edition (Bishop, 2003) and.85 with an existing Mandarin comprehension test (Chen, 2011). The English sentence recall task (The Redmond Sentence Recall Task, RSR, Redmond, 2005) is a standardized norm-referenced measure and consists of 16 sentences targeting the English past tense and passive construction. The norm was based on 782 typically-developing monolingual English-speaking children ages 5 to 9 years 5 months^1^. The RSR has a test-retest reliability of.95. Concurrent validity is.70 with the Test of Early Grammatical Impairment (Rice and Wexler, 2001) and.80 with the Clinical Evaluation of Language Fundamentals-4th Edition (Semel et al., 2003). The Mandarin sentence recall task (Wang et al. unpublished) was designed to be parallel to the RSR and capture the development of children 4–9 years of age. The task consists of 16 sentences targeting the Mandarin aspect markers, passive construction, relative clauses, and classifiers. Test-retest reliability is presently unavailable. Concurrent validity is.43 with mean length of utterance and.59 with a grammar composite score derived from spontaneous language samples based on data from monolingual Mandarin-speaking children (Wang et al. unpublished).

Parents filled out a questionnaire adapted from the Alberta Language Environment Questionnaire (ALEQ, Paradis, 2011) that collected information on language use at home, and richness of English and Mandarin use. In addition to these main language environment variables, the ALEQ also allowed us to collect information on children’s onset of language exposure, the parents’ education level, their mother tongue (i.e., language spoken growing up), and their self-ratings of spoken English proficiency. The ALEQ has been used with Chinese immigrant families in Canada (Paradis, 2011) and the United States (Song et al., 2021). We used the adaptation completed by Song et al. (2021), who translated the ALEQ into Mandarin Chinese and demonstrated significant associations between home language environment and language development trajectories in Chinese-English dual language learners.

Parents also filled out a brief questionnaire adapted from the Inventory to Assess Language Knowledge (ITALK, Peña et al., 2018). The Mandarin adaptation involved several rounds of translation and revision and the incorporation of Mandarin-specific examples to explain the rating scale. The Mandarin ITALK has been used in previous studies of Mandarin-English bilingual children (Sheng et al., 2011, 2014) as well as monolingual Mandarin-speaking children (Sheng et al., 2020). English and Mandarin oral language proficiency was rated on a 0–5 scale in the domains of vocabulary, sentence length, speech intelligibility, listening comprehension, and grammatical proficiency. Scores were averaged across the five domains to obtain an overall proficiency rating for each language.

The Primary Test of Non-verbal Intelligence (PTONI, Ehrler and McGhee, 2008) was used to measure children’s non-verbal IQ. The test requires children to look at displays of pictures and abstract patterns and point to the one that differs from the others. The PTONI was normed in a culturally diverse sample of 1,010 children from 38 United States.

### Procedures

**FIGURE 1:**
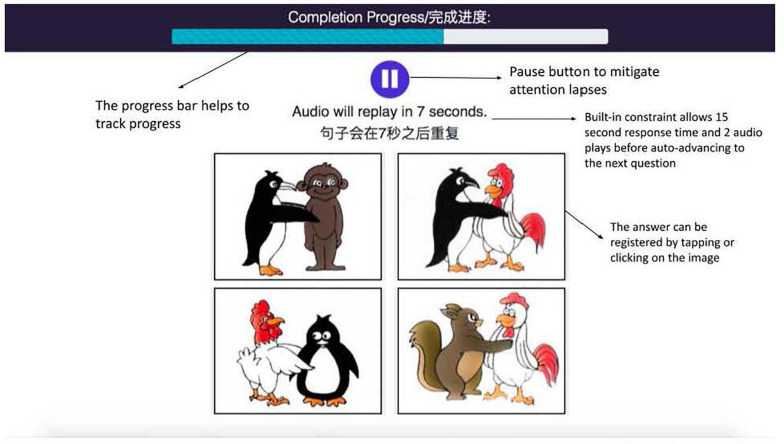
Illustration of a test item from the MECOLAB interface. The child hears the audio “the monkey is hugged by the penguin.”

The sentence comprehension task utilizes a sentence-picture matching format. Children have to match the auditorily presented sentence to one of the pictures on the screen. The number of picture choices varies from two to four depending on the test item. The task is hosted on an automated web-based interface called “Mandarin English Child Online Language Assessment Bank” (MECOLAB) designed by our lab (Du et al., 2020). The task began with the following instruction in English: “*Welcome. You will hear some sentences, and you will find the right picture. Let’s try it out. Are you ready*?,” and in Mandarin: “
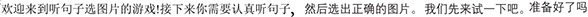
?” Two practice items were presented to familiarize children with the testing format. All children understood the instruction and were able to select their answers either independently or with the help of an adult. See Figure 1 for an illustration of the interface and a test item.

For the pre-COVID group, the items were presented on a DELL laptop computer with a 15-inch screen placed at roughly 1.5 feet away from the child. The audio was played at about 65dB through the computer’s built-in audio. For the COVID group, the tasks were administered through the Zoom platform. An online testing preparation sheet, which highlights equipment and environment requirements, was sent to the family the day before the scheduled appointment. The test items were presented on the child’s home computer or iPad, placed between 1 to 2 feet away from the child depending on location and setup. Parents were asked to set the computer’s volume to a comfortable level during an instructional video before the task began. The experimenter sent the link to the task website through the chat function, and the parent was asked to open the link and share their screen. If the child was unable to use a mouse to select their answers, they could point to the item on the screen and have the parent click for them.

The sentence recall tasks were presented on PowerPoint slides with each sentence pre-recorded on a separate audio file. The task began with the following instruction in English: *“Listen. I am going to say some sentences. After I have finished, I want you to say exactly what I have said. Say the same thing. Let’s try a sentence. Are you ready?,”* and in Mandarin: “
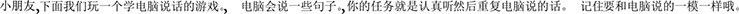
!” One English practice item and two Mandarin practice items were presented to familiarize the child with the sentence speed and task goals. All children understood the instruction and produced responses without parental assistance. For the pre-COVID group, the items were presented from a DELL laptop using the same setup as in the comprehension task. For the COVID group, items were presented *via* the screen share function on the Zoom platform from the experimenter’s computer.

Each child was seen over four weekly testing sessions to complete the larger study. In the first session, following the consenting process and collection of parent questionnaires, the English and Mandarin comprehension tasks were given in counterbalancing order across children. Children were able to take breaks in between English and Mandarin tasks. The English and Mandarin sentence recall tasks were administered in either the second or the third testing session, in counterbalancing order across children. The PTONI was administered along with the English sentence recall task.

### Data Analysis

Language use variables were calculated using the formula provided in the ALEQ (Paradis, 2011). Language use at home was calculated from a set of questions that asked about language use between the target child and the child’s mother, father, sibling(s), other primary caregiver(s), and adult relative(s) in the home. Each question uses a 5-point scale: 4 = English almost always/Mandarin almost never, 3 = English usually/Mandarin seldom, 2 = English 50%/Mandarin 50%, 1 = English seldom/Mandarin usually, and 0 = English never/Mandarin always. Because the number of family members varied across children, a proportion score was derived by adding up the total and dividing it by the number of questions that elicited a response multiplied by 4. The language richness scores were calculated from a set of questions that queried the frequency of literacy and language activities (e.g., reading, computer use, watching TV, storytelling, and singing) in each language, the frequency and language use of extra-curricular activities, the frequency of attending home language school programs, and the language spoken between the child and their friends. Questions on the frequency of language-related activities were answered using a 3-point scale: 2 = almost every day/every day, 1 = at least once a week, and 0 = almost never/never. Language spoken with friends was reported on a 5-point scale as described above. Again a proportion score was derived to reflect the number of questions that elicited responses.

Sentence comprehension responses were automatically registered by the computer and coded as correct or incorrect. Sentence recall responses were audio- or video-recorded and transcribed verbatim by trained research assistants who are native speakers of the respective languages. In both English and Mandarin, sentences were scored on a scale of 0–2 based on number of errors. Any word (in English) or syllable (in Mandarin Chinese) that was changed, added, substituted, or omitted was counted as one error. Each transposition that changed the meaning of the sentence was counted as two errors. Each transposition that did not change the meaning of the sentence was counted as one error. Dialectical differences and repetitions of words (or syllables), as in disfluencies or stuttering, were not counted as errors. Using the appropriate contracted or non-contracted form of an English verb (e.g., “did not” and “didn’t”) were considered interchangeable and did not count as an error. Sentences were given a score of 2 if they were repeated without errors, a score of 1 if they had 1 to 3 errors, and a score of 0 if they had 4 or more errors. The total score for the 16 sentences was summed, with a maximum score of 32 points.

Twenty percent of the sentence recall recordings in each language were transcribed by a second research assistant for reliability check. Reliability was calculated by dividing the number of consistent transcriptions by the number of total transcriptions. Consistency was counted on the basis of syllable in Mandarin and word in English. Interrater reliability was 97% for English transcription and 98% for Mandarin transcription. Scoring reliability was checked by having an additional research assistant independently score 20% of the responses. Interrater reliability was 91% for English scoring and 95% for Mandarin scoring. All inconsistencies were resolved through discussions.

To answer the first question, we compared language use at home, English richness, and Mandarin richness scores between the two samples using the Mann-Whitney U test, since the variables were not normally distributed. To answer the second question, we conducted two mixed-effect analysis of variance (ANOVA) with group as the between-subject variable, language as the within-subject variable, and comprehension and production task scores as the dependent measures. The English comprehension and production data were not normally distributed. However, ANOVA is robust to violations of the normal distribution assumption (Schmider et al., 2010). To answer the third question, we used the Spearman rank order correlation to examine the correlations between the three language use variables (i.e., language use at home, English richness, Mandarin richness) and the four language outcome measures in the pooled group and in each group. Before proceeding, we conducted a sensitivity analysis (Perugini et al., 2018) to determine what kind of effect sizes could be detected given the current sample size. With a sample of 19 children in each group and alpha set to be.05, we are at 80% power to detect a minimum effect size of.93 for *t*-tests (Cohen’s *d*) or a minimum correlational coefficient of.44. With a sample of 19 in a single group and alpha of.05, we are at 80% power to detect a minimum correlational coefficient of.60. For between-within two way ANOVA, we are at 80% power to detect a minimum effect size of *f* = 0.39 for the between-group factor, *f* = 0.26 for the within-group factor, and an *f* = 0.26 for the interaction. All estimates are based on two-tailed tests. All analyses except for one were conducted in R (R Core Team, 2020). The exception was the sensitivity analyses for ANOVA, which were conducted in G^∗^Power (Faul et al., 2007).

## Results

### Language Use

As shown in Table 1, the two groups did not differ in language use at home. Nor did they differ in English richness. On the other hand, Mandarin richness score was significantly higher in the COVID group than the pre-COVID group. According to Wilcoxon matched pairs test, both groups had higher English than Mandarin richness scores, pre-COVID: *T* = 1.00, *z* = 3.78, *p* < 0.001; COVID: *T* = 37.00, *z* = 2.33, *p* = 0.02. To better understand how Mandarin richness differed in pre-COVID and COVID samples, we compared the average score between groups for individual questions that made up the Mandarin richness composite score using Mann-Whitney U tests. We also performed this *post hoc* exploratory analysis on the language-use-at-home questions because although the *p* level (0.10) was not significant, it was low. Of the 10 language-use-at-home questions, six had sample sizes of at least 10 per group. This was because only a few children had non-parent caregivers or adult relatives in the home. Only the six questions (i.e., language use between child and mother, child and father, and child and sibling) with at least 10 responses per group were analyzed.

This exploratory analysis revealed differences in the frequency of Mandarin-related activities and the language spoken between the target child and their friends and between the target child and their parents. Specifically, the COVID group showed higher frequency than the pre-COVID group on the following items: using computers and other electronic devices for language-related activities (e.g., web surfing, playing games, and listening to stories) (pre-COVID: *M* = 0.22, *SD* = 0.55; COVID: *M* = 0.84, *SD* = 0.90, *W* = 105.50, *p* = 0.02); watching Mandarin shows, movies, or videos (pre-COVID: *M* = 0.44, *SD* = 0.51; COVID: *M* = 1.11, *SD* = 0.81, *W* = 93.00, *p* = 0.01); and attending Mandarin language classes (pre-COVID: *M* = 0.50, *SD* = 0.52; COVID: *M* = 1.05, *SD* = 0.78, *W* = 92.00, *p* = 0.03). Moreover, the COVID group used less English (*M* = 2.58, *SD* = 1.17) and more Mandarin (*M* = 1.42, *SD* = 1.17) with their friends in comparison to the pre-COVID group (English: *M* = 3.53, *SD* = 0.70; Mandarin: *M* = 0.47, *SD* = 0.70), *W* = 263.50, *p* = 0.01. Mother-to-child language use was significantly different between groups: the mothers of the COVID group (*M* = 0.65, *SD* = 0.75) spoke less English and more Mandarin to the target child than the mothers in the pre-COVID group (*M* = 1.00, *SD* = 0.56), *W* = 251.00, *p* = 0.02. Finally, there were significant differences in child-to-mother (COVID group: *M* = 0.79, *SD* = 0.79; pre-COVID group: *M* = 1.58, *SD* = 1.12, *W* = 253.50, *p* = 0.03) and child-to-father language use in the same direction (COVID group: *M* = 0.74, *SD* = 0.73; pre-COVID group: *M* = 1.58, *SD* = 1.26, *W* = 251.50, *p* = 0.03). Individual level data for input related questions can be found in the Supplementary Materials.

### Language Performance

**FIGURE 2:**
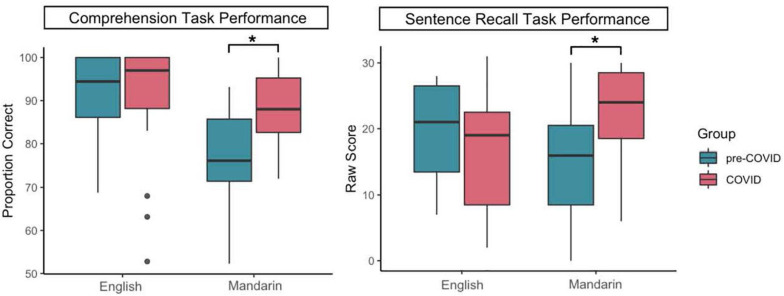
Performance on the comprehension and the sentence recall task as a function of group and language.

For comprehension, there is a main effect of language, *F*(1, 36) = 23.53, *p* < 0.001, *f* = 0.82. Children were more accurate in English (*M* = 0.91, *SE* = 0.02) than Mandarin (*M* = 0.83, *SE* = 0.02). The interaction between language and group was also significant, *F*(1, 36) = 15.03, *p* < 0.001, *f* = 0.64. *Post hoc* tests showed that the pre-COVID (*M* = 0.92, *SD* = 0.08) and COVID (*M* = 0.90, *SD* = 0.14) groups were comparable in English comprehension, *p* = 0.74, *d* = 0.18; however, the COVID group (*M* = 0.88, *SD* = 0.09) was more accurate than the pre-COVID group (*M* = 0.77, *SD* = 0.12) in Mandarin comprehension, *t* = 3.52, *df* = 36, *p* = 0.001, *d* = 1.04. *Post hoc* Wilcoxon matched pair tests showed that within the pre-COVID group, English comprehension performance (*M* = 0.92, *SD* = 0.08) was significantly better than Mandarin comprehension (*M* = 0.77, *SD* = 0.12), *T* = 5.00. *Z* = 3.62, *p* < 0.001. Within the COVID group, comprehension performance was comparable between English (*M* = 0.90, *SD* = 0.14) and Mandarin (*M* = 0.88, *SD* = 0.09), *p* = 0.30. These patterns are illustrated in the left panel of Figure 2^2^.

For production, there were no main effects of language and group, but the interaction between language and group was significant, *F*(1, 36) = 12.00, *p* = 0.001, *f* = 0.58. While the pre-COVID group (*M* = 19.84, *SD* = 6.84) and the COVID group (*M* = 16.21, *SD* = 8.34) were comparable on English sentence recall, *p* = 0.13, *d* = 0.48; the COVID group (*M* = 22.42, *SD* = 6.59) was more accurate than the pre-COVID group (*M* = 14.84, *SD* = 8.36) on Mandarin sentence recall, *t* = 3.10, *df* = 36, *p* = 0.004, *d* = 1.01. Within the pre-COVID group, English recall score (*M* = 19.84, *SD* = 6.84) was significant higher than Mandarin (*M* = 14.84, *SD* = 8.36), *T* = 33.50, *Z* = 2.04, *p* = 0.04. Within the COVID group, English (*M* = 16.21, *SD* = 8.34) was significant lower than Mandarin (*M* = 22.42, *SD* = 6.59), *T* = 36.50, *Z* = 2.13, *p* = 0.03. These patterns are illustrated in the right panel of Figure 2.

### Relationship Between Language Use and Performance

All results are displayed in Table 2. It is worth pointing out that the sample size was limited and some of the correlations were lower than the minimum *r* values obtained in the sensitivity analysis. These correlation results could be unstable. For the pooled group, out of 12 correlations, five were significant: language use at home was correlated with English sentence recall (*rho* = 0.43, *p* = *0.01*) and Mandarin sentence recall (*rho* = −0.41, *p* = 0.01). This is because higher language use at home score means higher English use. English richness was correlated with English sentence recall (*rho* = 0.33, *p* = 0.04), and Mandarin richness was correlated with both Mandarin comprehension (*rho* = 0.41, *p* = 0.01), and Mandarin sentence recall (*rho* = 0.52, *p* = *0.001*). Three out these five significant correlations remained significant and in the same direction for the COVID group, yet none of the correlations was significant in the pre-COVID group. In other words, the association between language use and language outcome was stronger in the COVID group than the pre-COVID group.

**TABLE 2:** Summary of Spearman’s rank order correlation matrix between language use and language performance measures.

	Language use at home		English richness		Mandarin richness	
	*rho*	*p*	*rho*	*p*	*rho*	*p*
Pooled						
English comprehension	0.23	0.17	−0.11	0.51	−0.16	0.34
Mandarin comprehension	−0.08	0.63	−0.10	0.57	**0.41**	0.01*
English recall	**0.43**	0.009**	**0.33**	0.04*	−0.30	0.07
Mandarin recall	−**0.41**	0.01*	−0.05	0.75	**0.52**	0.001**
Pre-COVID						
English comprehension	0.06	0.80	−0.32	0.18	−0.19	0.44
Mandarin comprehension	−0.12	0.63	−0.17	0.50	0.20	0.44
English recall	0.20	0.46	−0.08	0.74	0.06	0.83
Mandarin recall	−0.42	0.07	0.04	0.87	0.16	0.51
COVID						
English comprehension	0.38	0.11	0.03	0.91	−0.08	0.74
Mandarin comprehension	0.23	0.36	−0.06	0.82	0.14	0.56
English recall	**0.55**	0.01*	**0.68**	0.001**	−0.34	0.15
Mandarin recall	−0.23	0.35	−0.24	0.32	**0.62**	0.004**

Language use at home varies from 0 to 1, with 0.5 meaning equal English and Mandarin use at home, values > 0.5 meaning more English than Mandarin, and values < 0.5 meaning more Mandarin than English. Language richness scores vary from 0 to 1, with higher scores meaning richer language use. *p < 0.05; **p < 0.01. Bolded values are statistically significant.

Because exploratory analyses under 3.1 revealed group differences on seven language use questions (i.e., use of computer for Mandarin language activities, watch Mandarin TV shows, taking (online) Mandarin classes, language use with friends, mother-to-child language use, child-to-mother language use, child-to-father language use), we ran additional correlations between these seven variables and the four performance variables in the pooled group. Given the large number of analyses, we used a conservative *p* value of.0017 (0.05 divided by 28 analyses). Three correlations were significant: more English use between the child and their friends was associated with higher English sentence recall scores (*rho* = 0.52, *p* < 0.001), more child-to-mother Mandarin use was positively associated with Mandarin sentence recall (*rho* = 0.53, *p* < 0.001), and more child-to-father Mandarin use was positively associated with Mandarin sentence recall (*rho* = 0.51, *p* = 0.0011).

## Discussion

Using data from matched samples of bilingual children, we were able to closely examine language use patterns and bilingual language performance, and establish associations between the two against the backdrop of COVID-19 induced language environment changes. In early school age Mandarin-English bilinguals, language use in the home was not significantly different between samples tested before and during the pandemic. This lack of difference may be due to low power because we were equipped to detect large effects only. Exploratory analyses indicated more use of Mandarin between the child and their parents in the COVID sample than the pre-COVID sample. These findings align with a recent report (Serratrice, 2020) of elevated use of the home language in bilingual children of comparable age during lockdown.

While total English richness scores were similar between the two groups, Mandarin richness score was elevated in the COVID group compared to their peers tested before the pandemic. Four out of eight questions that probed Mandarin richness showed group differences, indicating more frequent use of Mandarin with friends, and more frequent use of digital device for Mandarin language activities. Drastically increased screen time was reported in large-scale datasets of American families after the onset of the pandemic and this increase was attributed to family stress, in particular, decreased adult caretaker availability (Hartshorne et al., 2021). It is unclear whether the increased use of digital device for Mandarin related activities in the current sample was an intentional decision by the parents to boost the home language, or a fortuitous by-product of “screens as the babysitter” (Hartshorne et al., 2021, p.1). Despite the higher Mandarin richness scores in the COVID group, in both groups of children, English richness was still significantly higher than Mandarin richness. This is consistent with a recent study of young Chinese-English bilinguals (Song et al., 2021) and suggests that intentional language enrichment activities are still more difficult to implement in bilingual’s home language due to the lack of learning materials, the educational focus on English, and children’s own preference.

Turning to children’s performance, we found similar patterns between comprehension and production modalities. In both modalities, children in the COVID group showed an advantage in the home language but did not lose ground in English in comparison to the children tested before the pandemic. Moreover, the pre-COVID sample was clearly English-dominant in both modalities, whereas the COVID sample was balanced in comprehension and Mandarin-dominant in the production modality. The pre-COVID patterns replicated previous studies of United States Mandarin-English bilingual children from middle-class background (Sheng et al., 2011, 2014; Hao et al., 2019). Taken together, these findings suggest a home language boost in both comprehension and production, accompanied by relatively preserved English comprehension and production skills in the COVID group.

This pattern of overall gains is consistent with recent reports of first and second language learning during the COVID era (Hopp and Thoma, 2020; Kartushina et al., 2021). Hopp and Thoma (2020) compared cross-sectional samples of German primary school learners of English: one group experienced 15 weeks of foreign language instruction interruption secondary to COVID-induced school closure and curricular reduction, the other group was assessed the year prior and had continuous English instruction. Both groups were assessed three times over an academic year. The authors did not find any negative impact of temporary instructional suspension on foreign language vocabulary and grammar. The group that experienced instruction suspension made as much gains in English as the group that had continuous instruction. Kartushina et al. (2021) conducted a large-scale multinational study of vocabulary development in monolingual infants and toddlers using various versions of the Communicative Development Inventories (Fenson et al., 2007). They found that children gained more words than expected (based on normative data) during lockdown, a result that could be explained by either the parents’ increased awareness of their children’s vocabulary knowledge or more intense caregiver-child interactions during lockdown. Children who had less passive screen exposure and whose caregivers read more to them gained more words.

Although the COVID group did not lose ground in English sentence recall performance relatively to peers tested before the pandemic, the within-group cross-language comparisons suggested that the extent of dominance shift was greater in production than in comprehension. This is consistent with previous studies that indicated more rapid language dominance shifts in the expressive modality than receptive modality and highlights the resiliency of receptive language to changes in the language environment (e.g., Pham and Kohnert, 2014).

The correlation analyses helped connect language proficiency to language use. Production skills in both languages were related to several language use variables. Both the amount and the contextual diversity of language use were related to performance on both English and Mandarin sentence recall. In particular, language use between the child and their friends, and between the child and their parents stood out as having strong relationships with children’s production facility. Peer language use is known to play important roles in bilingual language development (Chesterfield et al., 1983; Kan et al., 2020). During an ongoing global pandemic, opportunities interacting with non-family members may be extremely limited. Hence, conversations with peers outside the family may be especially salient in shaping children’s English language production. Within the home environment, both mothers and fathers appeared to be influential in shaping children’s bilingual language production skills. All children in the current sample resided in two-parent households with both parents being native speakers of Mandarin. These findings substantiate the importance of “Family Language Policy” in children’s developing bilingual proficiency (De Houwer and Bornstein, 2016). In comparison to the robust relationships between language use and production skills, we found only one correlation between language use and comprehension skills, in the form of a positive correlation between Mandarin richness and Mandarin comprehension in the pooled sample. However, this correlation ceased to exist when the groups were separated. Also of note is the finding that the correlations were far more robust in the COVID group than the pre-COVID group. We speculate that this difference could be partially due to the features of the language environment questions: the ALEQ (Paradis, 2011) was designed to focus on language use at home. While the language input variables in the current study could not fully capture the language environment experienced by the pre-COVID sample (e.g., school, community), because of home confinement, these variables were able to better encapsulate the language experiences of the COVID group.

In summary, the COVID-19 pandemic presents an unprecedented opportunity to study the effect of language environment modification in bilingual language use and proficiency. Against this backdrop, we found greater and contextually richer use of the bilinguals’ home language. Extrapolating from the cross-sectional data, our findings suggested that a shift from English dominance to balanced skills in the comprehension modality and a shift toward Mandarin dominance in the production modality may be taking place. Along with other conditions of language environment change (Glennen, 2002; Kohnert and Bates, 2002; Krakow et al., 2005; Sheng et al., 2011, 2014; Dewey et al., 2012; Rojas and Iglesias, 2013; Pham and Kohnert, 2014; Sheng, 2014; Di Silvio et al., 2016), these findings add to the body of literature that supports an usage-based account of bilingual language acquisition (Wulff and Ellis, 2018; Oppenheim et al., 2020).

Before closing, we must stress that the present study was a small-scale retrospective study of existing data. As such, we could not rule out other potential explanations for the observed patterns. For example, it is possible that the COVID group’s enhanced Mandarin performance relative to the pre-COVID group was due to preexisting differences in Mandarin skills driven by preexisting differences in their Mandarin environment. As the data were not collected to address COVID-related environmental changes, we did not ask the parents about differences in language use habits before and during COVID. For instance, information on parental language switching tendencies was not collected but could influence bilingual language learning (Byers-Heinlein, 2013). Also, we did not collect information on schooling status for the pre-COVID group, although the predominant mode was in-person in the United States prior to COVID. The schooling mode among the COVID group differed from child to child, which could have increased the variability in the data and impacted the results. In addition, the testing period for the pre-COVID group covered winter, spring, and summer (January to August of 2019), whereas the testing period for the COVID group covered summer, fall, and winter (June 2020 to January 2021). This time of year difference could also have implications for family language use habits and child language performance outcomes. All of these factors should be considered in the design of future studies.

Because of the small sample size and lack of power, we did not correct the *p* level for a majority of the analyses. This could have increased the chance of false positives. Moreover, many of the statistically significant correlations were lower than the minimum *r* values.44 for the pooled sample and.60 for a single group as indicated by the sensitivity analyses. This suggests that future studies with a similar sample size may not reliably detect the same effects.

These limitations notwithstanding, the current findings invite more prospective studies on the effect of COVID-related environmental modifications on children’s language development. Many questions remain. For example, how generalizable are these results to other groups of bilinguals who may not have the same kinds of resources as the current sample? How long-lasting are the home language gains? Will the gains erode and dissipate quickly in the post-COVID era? Longitudinal studies of these bilingual children in the future will provide insights into the durability of these effects of environmental modifications.

## Data Availability Statement

The original contributions presented in the study are included in the article/Supplementary Material, further inquiries can be directed to the corresponding author/s.

## Ethics Statement

The studies involving human participants were reviewed and approved by the Institutional Review Board of the University of Delaware. Written informed consent to participate in this study was provided by the participants’ legal guardian/next of kin.

## Author Contributions

LS secured the funding for the project, and conceptualized and drafted the manuscript. DW, CW, and LH collected the data, and transcribed and coded the responses. XL transcribed and coded Mandarin sentence recall data. DW and PS performed statistical analyses and produced some of the graphics. All authors participated in the editing processes and approved the final version of the manuscript.

## Conflict of Interest

The authors declare that the research was conducted in the absence of any commercial or financial relationships that could be construed as a potential conflict of interest.

## Footnotes

1 We had four children who were 4 years old in our sample. Their scores ranged from 6 to 22, whereas in the whole sample the scores ranged from 2 to 31. These distributions suggested that the items were developmentally appropriate for children who were outside of the age norm.

2 Using 3 SDs from the mean as the cutoff, we found one outlier from the COVID group on the English comprehension measure who scored 3.33 SD below the mean. We re-ran the ANOVA with this child and their match removed. The results did not change. No outliers were detected for the production measure.
