# Supplementary material for: The Bilingual Home Language Boost Through the Lens of the COVID-19 Pandemic
Source: Front Psychol. 2021 Jul 20;12:667836. doi: 10.3389/fpsyg.2021.667836 (PMC8329553; doi:10.3389/fpsyg.2021.667836)
Supplement: Supplementary file 1 [file Data_Sheet_1.PDF]

Table 1. Description of target types. Each type includes 8 items.

English Target	Literature & Published Tests	Mandarin Target	Literature & Published Tests
Prepositional phrases	*QUILS*[1]; *TROG*[2]	Prepositional phrases	*DREAM*[3]; Miao & Zhu[4]
WH questions	*QUILS*[1]; Deevy et al[5]	WH questions	*DREAM*; Wong et al[6]
Noun plurals	*TROG*[2]; Jia[7]; Sheng et al[8]	Noun classifiers	Erbaugh[9]; Kan & Sheng[10]
Quantifiers	Crain[11]; Katsos et al[12]; *DELV*[13]	Quantifiers	Zhou & Gao (2009)[14]; Zhou & Crain (2011)[15];
Passive sentences	*DELV*[13]*; TROG*[2]	Passive sentences	Ji et al[16], Sheng et al[8]
3rd personal singular	Johnson et al[17]; Pawlowska et al[18]	BA sentences (i.e., non-canonical active sentences)	Ji et al[16]
Conjunctions	*QUILS*[1]; *TROG*[2]	Conjunctions	Miao & Zhu[4]
Relative clauses	Frizelle et al[19]; *TROG*[2]	Relative clauses	Hu et al[20]

Note. *QUILS*: Quick Interactive Language Screener[1]. *TROG*: Test of Receptive Grammar[2]. *DREAM*: Diagnostic Receptive and Expressive Assessment of Mandarin[3]. *DELV*: Diagnostic Evaluation of Language Variation[13]. The *DREAM* is not readily accessible. Information is based on a small number of sample items in Liu et al[54]. It is likely that other Mandarin targets are also represented in the *DREAM*. The target elements are not always the same between English and Mandarin because cross-linguistic differences in language structure lead to different elements being more sensitive to impairment.

References for Table 1

1. Golinkoff, R. M., De Villiers, J. G., Hirsh-Pasek, K., Iglesias, A., Wilson, M. S., Morini, G., & Brezack, N. (2017). *User's Manual for the Quick Interactive Language Screener (QUILS): A Measure of Vocabulary, Syntax, and Language Acquisition Skills in Young Children*. Paul H. Brookes Publishing Company.

2. Bishop, D. V. M. (2003). The test for reception of grammar, version 2 (TROG-2). London: Pearson.

3. Ning, C. Y., Liu, X. L., & de Villiers, J. G. (2014). The diagnostic receptive and expressive assessment of Mandarin. *Dallas, TX: Bethel Hearing and Speaking Training Center*.

4. Miao, X., & Zhu, M. (1992). Language Development in Chinese children. In H.C. Chen, & O.J.L. Tzeng (Eds.), *Language processing in Chinese* (pp. 237-276). Amsterdam: Elsevier Science Publishers.

5. Deevy, P., & Leonard, L. B. (2004). The comprehension of wh-questions in children with specific language impairment. *Journal of Speech, Language, and Hearing Research, 47*(4), 802-815.

6. Wong, A. M. Y., Leonard, L. B., Fletcher, P., & Stokes, S. F. (2004). Questions without movement: A study of Cantonese-speaking children with and without specific language impairment. *Journal of Speech, Language, and Hearing Research*, *47*(6), 1440-1453.

7. Jia, G. (2003). The acquisition of the English plural morpheme by native Mandarin Chinese-speaking children. *Journal of Speech, Language, and Hearing Research*, *46*(6), 1297-1311.

8. Sheng, L., Yang, M., Peña, E., Bedore, L., Li, F., & Du, Y. (2016, June). Profiles of language impairment in Mandarin-English bilingual children: A pilot study. Poster presented at the 37th annual Symposium on Research in Child Language Disorders, Madison, Wisconsin.

9. Erbaugh, M. S. (2006). Chinese classifiers: their use and acquisition. In P. Li, L.H. Tan, E. Bates, & O.J.L. Tzeng (Eds.), The handbook of East Asian Psycholinguistics: Chinese, (pp. 39-51). Cambridge: Cambridge University Press.

10. Kan, P.F., & Sheng, L. (2017, June). Noun Classifier Development in Sequential Bilingual Preschool Children. Poster presented at the 11th International Symposium on Bilingualism, Limerick, Ireland.

11. Crain, S. (2017). Acquisition of Quantifiers. *Annual Review of Linguistics*, *3*(1), 219–243. https://doi.org/10.1146/annurev-linguistics-011516-033930

12. Katsos, N., Cummins, C., Ezeizabarrena, M.-J., Gavarró, A., Kuvač Kraljević, J., Hrzica, G., … Noveck, I. (2016). Cross-linguistic patterns in the acquisition of quantifiers. *Proceedings of the National Academy of Sciences*, *113*(33), 9244–9249. https://doi.org/10.1073/pnas.1601341113

13. Seymour, H.N., Roeper, T.W., & de Villiers, J. (2003). *Diagnostic evaluation of language variation*. San Antonio, TX: The Psychological Corporation.

14. Zhou, P., & Gao, L. (2009). Scope processing in Chinese. *Journal of Psycholinguistic Research*, *38*(1), 11-24.

15. Zhou, P., & Crain, S. (2011). Children's knowledge of the quantifier dou in Mandarin Chinese. *Journal of psycholinguistic research*, *40*(3), 155-176.

16. Ji, Y., Zheng, L. & Sheng, L. (2018). Acquisition of noncanonical word orders in Mandarin Chinese. Manuscript under review.

17. Johnson, V. E., de Villiers, J. G., & Seymour, H. N. (2005). Agreement without understanding? The case of third person singular/s. *First Language*, *25*(3), 317-330.

18. Pawłowska, M., Leonard, L. B., Camarata, S. M., Brown, B., & Camarata, M. N. (2008). Factors accounting for the ability of children with SLI to learn agreement morphemes in intervention. *Journal of Child Language*, *35*(1), 25-53.

19. Frizelle, P., O'Neill, C., & Bishop, D.V.M. (2017). Assessing understanding of relative clauses: a comparison of multiple-choice comprehension versus sentence repetition. *Journal of Child Language*, *44*, 1435-1457.

20. Hu, S., Gavarró, A., Vernice, M., & Guasti, M. T. (2016). The acquisition of Chinese relative clauses: contrasting two theoretical approaches. *Journal of Child Language*, *43*(1), 1-21.

I. Sample Items for Each Target Construct

English Target	Audio	Images
Prepositional phrases	The flower is under the table.	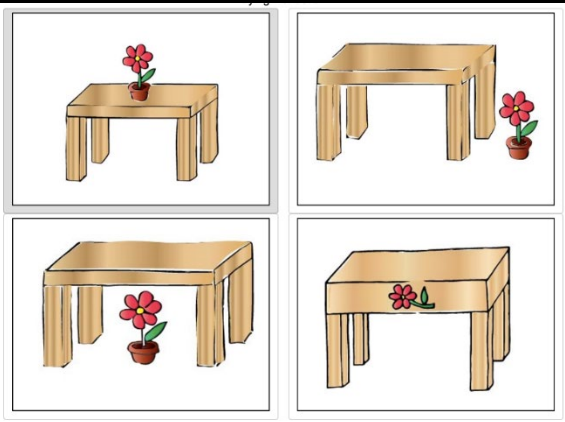
WH questions	Who is dressing the boy?	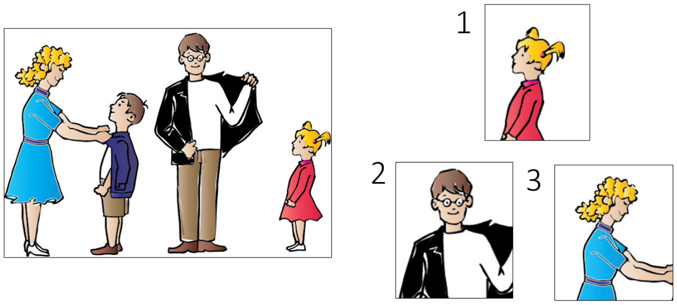
Noun plurals	The lady makes the cakes.	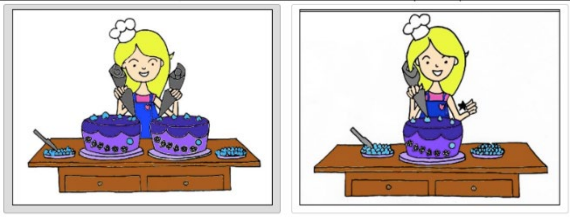
Quantifiers	Some frogs are holding an apple.	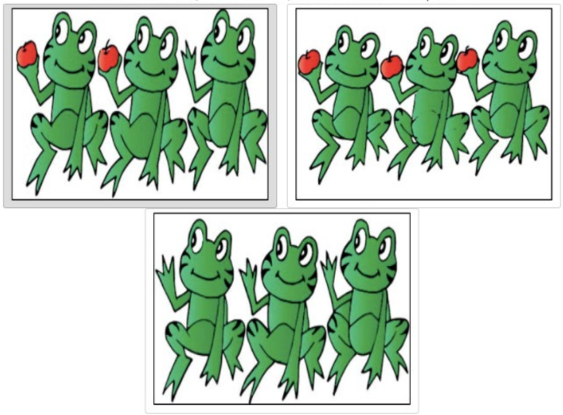

Passive sentences	The chicken is hugged by the penguin.	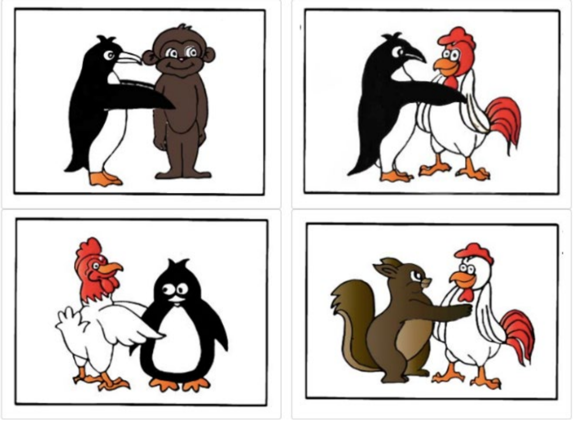
3rd personal singular	The duck swims.	1 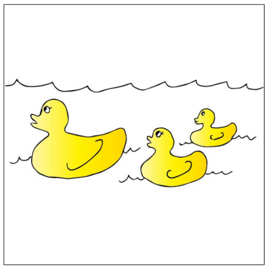 2 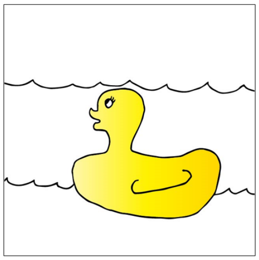
Conjunctions	The girl bought some apples before riding the bus.	1 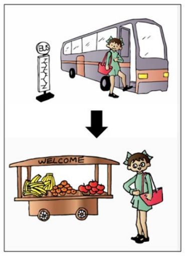 2 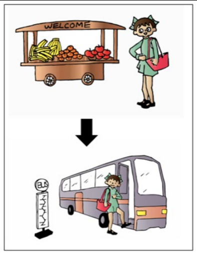
Relative clauses	The rabbit that is eating is hitting the cat.	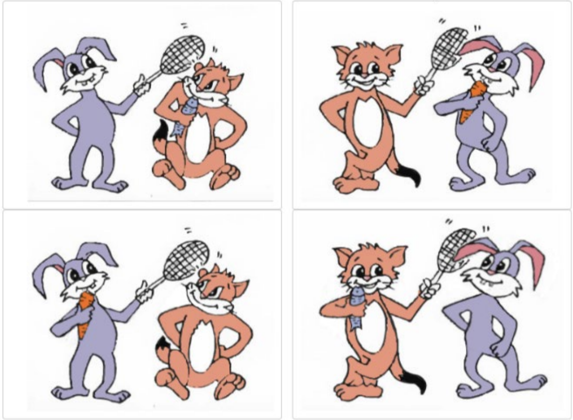
Mandarin Target	Audio Translation	Images

Prepositional phrases	长颈鹿在椅子后面	The giraffe is behind the chair.	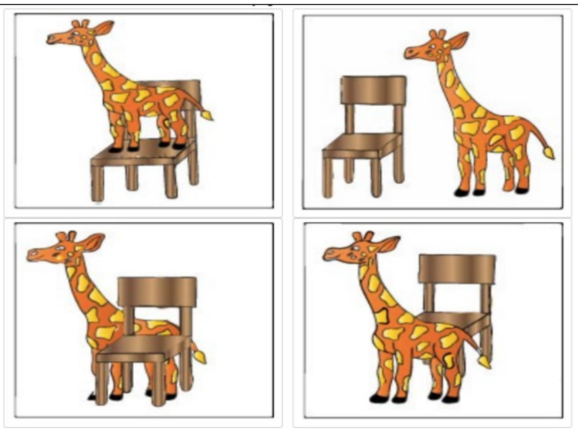
WH questions	谁在踢球?	Who is kicking the ball?	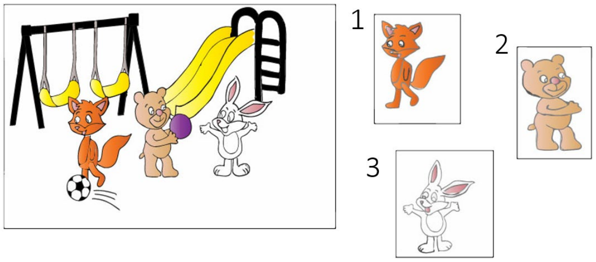
Noun classifiers	小狗说他要一张 something	Puppy says that he wants one *zhang* something.	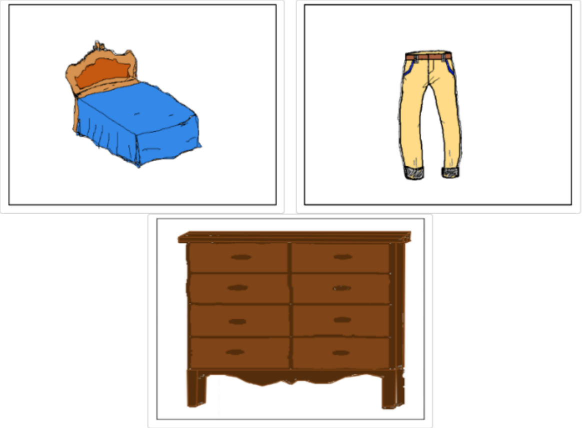
Quantifiers	有的小狗在啃骨头	Some dogs are biting the bone.	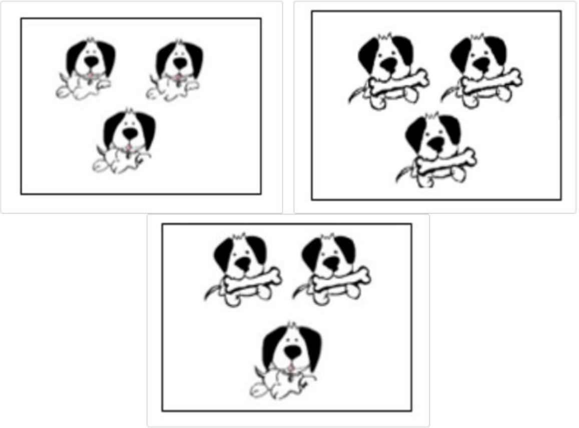

Passive sentences	乌龟被小猫绑着	The turtle is tied by the cat.	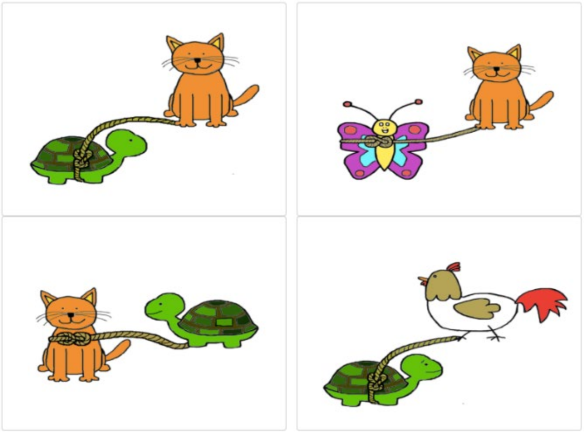
BA sentences (i.e., non-canonical active sentences that follows a Subject-Object-Verb order)	小鸡把青蛙抱着	The chicken is hugging the frog.	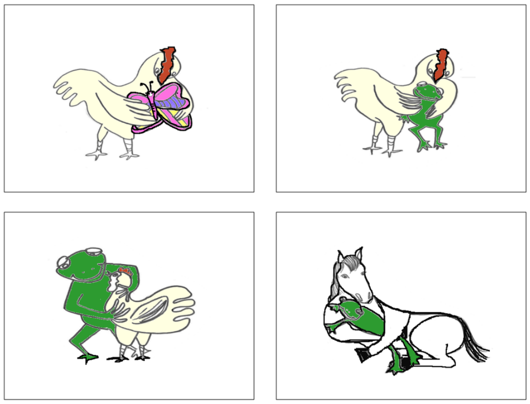
Conjunctions	小女孩在刷牙之后洗了脸	The girl washes her face after brushing her teeth.	1 2 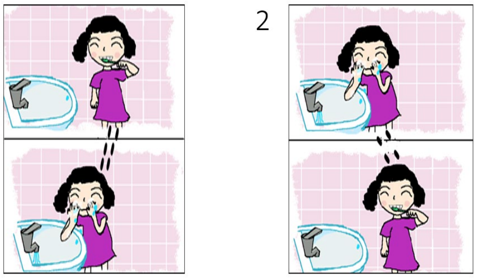
Relative clauses	亲奶奶的女孩在看书	The girl that is kissing the grandma is reading.	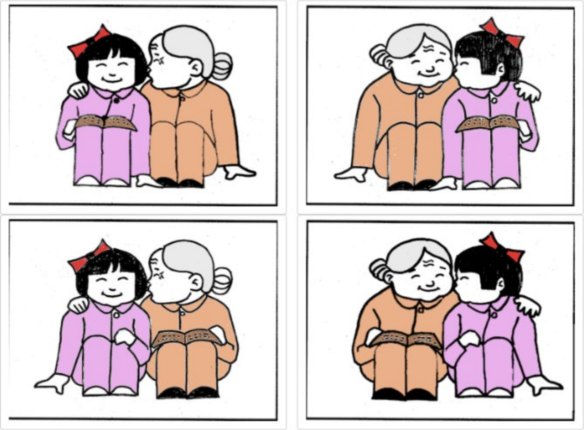
